# 2Statistically significant dependence of the Xaa-Pro peptide bond conformation on secondary structure and amino acid sequence

**DOI:** 10.1186/1472-6807-5-8

**Published:** 2005-04-01

**Authors:** Doreen Pahlke, Christian Freund, Dietmar Leitner, Dirk Labudde

**Affiliations:** 1 Forschungsinstitut für Molekulare Pharmakologie Robert-Rössle-Str. 10, D-13125 Berlin, Germany; 2 Biotechnologisches Zentrum Universität Dresden, AG Zelluläre Maschinen Tatzberg 47–51, D-01307 Dresden, Germany

## Abstract

**Background:**

A reliable prediction of the Xaa-Pro peptide bond conformation would be a useful tool for many protein structure calculation methods. We have analyzed the Protein Data Bank and show that the combined use of sequential and structural information has a predictive value for the assessment of the cis versus trans peptide bond conformation of Xaa-Pro within proteins. For the analysis of the data sets different statistical methods such as the calculation of the Chou-Fasman parameters and occurrence matrices were used. Furthermore we analyzed the relationship between the relative solvent accessibility and the relative occurrence of prolines in the cis and in the trans conformation.

**Results:**

One of the main results of the statistical investigations is the ranking of the secondary structure and sequence information with respect to the prediction of the Xaa-Pro peptide bond conformation. We observed a significant impact of secondary structure information on the occurrence of the Xaa-Pro peptide bond conformation, while the sequence information of amino acids neighboring proline is of little predictive value for the conformation of this bond.

**Conclusion:**

In this work, we present an extensive analysis of the occurrence of the cis and trans proline conformation in proteins. Based on the data set, we derived patterns and rules for a possible prediction of the proline conformation. Upon adoption of the Chou-Fasman parameters, we are able to derive statistically relevant correlations between the secondary structure of amino acid fragments and the Xaa-Pro peptide bond conformation.

## Background

The peptide bond has a partial double bond character which results in the plane arrangement of the six backbone atoms C^α^_(i-1)_, C'_(i-1)_, O_(i-1)_, N_(i)_, H_(i)_, C^α^_(i)_. The angles Ψ, Φ and Ω readily describe the arrangement of the six atoms in three-dimensional space: Ψ defines the angle of the N – C^α ^bond and Φ is given by the C^α^-C' bond of the same residue whereas the Ω angle is defined between the C' and N atoms of adjacent residues. For Ω only two conformations are energetically and sterically preferred. The trans conformation is defined by an Ω angle of 180° while the cis conformation ideally displays an Ω angle of 0°. The cis conformation occurs rarely in polypeptides because of the higher intrinsic energy compared to the trans conformation [1]. In contrast to other amino acids proline has a higher propensity for the cis conformation. This can be explained by the smaller energy difference between the cis and trans isomer which is 2 kcal/mol higher for the cis as compared to the trans imide bond. The functional relevance of the proline cis/trans equilibrium is supported by the existence of special enzymes called peptidyl-prolyl isomerases which catalyze the cis/trans isomerization of Xaa-Pro bond [2,3]. The action of these enzymes is thought to be important for the proper functioning of biological processes such as protein folding [4,5] and splicing [6]. In addition, cis prolines act possibly as molecular switches [7] and are frequently present in turn regions of water-soluble proteins [8].

Nuclear magnetic resonance (NMR) experiments have shown that the cis/trans ratio depends on the amino acid sequence adjacent to the proline. More specifically, a correlation has been found between the isomerization rate and the bulkiness of the side chain of the residue preceding proline. The isomerization rate becomes smaller as the bulkiness of the side chain increases. For example, aromatic residues cause an approximately tenfold reduction in isomerization rate in comparison to alanine [9]. Further NMR studies [10] demonstrated that the cis/trans ratio is influenced by the nature of the succeeding amino acid. Positively charged side chains seem to destabilize cis relative to trans whereas aspartate, asparagine and glycine stabilize the cis form.

Cis and trans isomers show different tendencies to be present in certain secondary structure elements: While the trans conformations can be found in all classes of secondary structure (in helices at the beginning or the end and in the center of helices causing a sharp kink of ≥ 20°) [11], the cis isomer is usually confined to bend and turn regions within proteins. However, a systematic analysis of these findings has been elusive.

The goal of our study is to derive statistically relevant propensities for predicting the relative occurrence of cis and trans proline conformations in proteins with respect to their sequential and structural properties.

For the analysis of the data set, different statistical methods such as calculation of the Chou-Fasman parameters [12] and occurrence matrices were used.

## Results and discussion

### Chou-Fasman parameters

**Figure 1:**
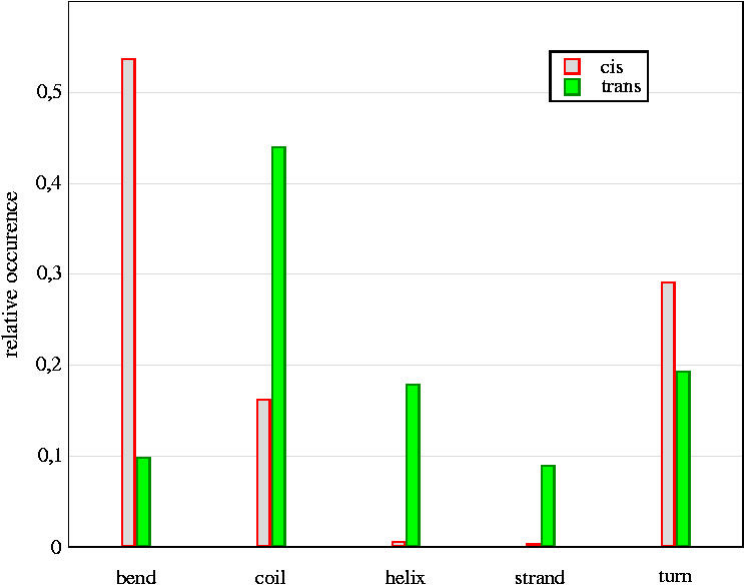
**Occurrence of secondary structure**. Relative occurrence of the secondary structure types compared for cis and trans prolines.

Figure (1) shows the comparison of the relative natural occurrence of the secondary structure types for cis and trans conformations of the Xaa-Pro peptide bond based on the PDB analyses (see Materials and Methods). Interestingly, cis prolines mostly occur in bend structures and almost never in helix or strand whereas trans prolines are mostly found in coil and to a smaller degree in turn, bend, helix or strand structures.

This observation is confirmed by analysis of the data with the modified Chou-Fasman parameters (equations 1–3 in the Methods section). The  values obtained from this analysis (Table (1)) show that the parameter values are close to one for the trans proline, indicating no significant preference for any given secondary structure type. In contrast, the  value is high for cis prolines within the bend structure type (4.249) while it is very small for helix (0.036) or strand (0.051) elements.

In addition, the Chou-Fasman parameters of the secondary structures of the two amino acids adjacent to proline (i-1 and i+1) are calculated and illustrated in Table (2). Similar to the results for the central proline position (Table (1)), the trans conformation of the proline is almost equally distributed amongst the different secondary structure types for these two positions (values close to 1). For cis prolines, the Chou-Fasman parameter at positions (i-1) and (i+1) show a strong bias against helical secondary structure, albeit not as strong as for the proline itself (0.086 and 0.321, respectively). The low propensity for the strand structure seen for the central cis proline is not observed for the preceding residue and is above 1 for the residue following proline. The preference for the bend structure is attenuated for the adjacent residues (2.054 and 1.263, respectively) as compared to the central cis proline (4.249). Interestingly, the propensity for the residue preceding cis proline to be part of a turn structure is higher than to be part of a bend structure (2.261 and 2.054, respectively). For the cis proline itself, the order is reverse.

### Occurrence matrix

A matrix of residue occurrence of the five-piece fragments (Table (3)) reveals the different preferences of residues adjacent to proline. On the left side all residue combinations with a trans proline in the mid-position are listed and on the right side all combinations with a cis proline in this position are reported. Rows indicate the amino acid types and columns contain the absolute and relative occurrences of the amino acids in the five-pieces fragments with proline in the central position.

Table (3) illustrates the significant changes of the natural occurrence of the amino acids at different positions. The calculated natural occurrences (column 2) correspond to the results by [13].

The observed relative occurrences were normalized in respect to the natural occurrence at each position for each amino acid type (Table (4)). The normalized occurrences for cis and trans (trans proline conformation Htrans and cis proline conformation Hcis) show the preference for certain amino acid sequence patterns. Amino acids with a relative occurrence greater one are shown in bold face. We considered a relative occurrence difference as almost equal (≈) if ΔH was smaller than 0.05. ΔH values larger than 0.5 were of particular significance (>>). The following observations can be made: For the normalized occurrences (Htrans/cis) of the trans conformations at the position i-2 the amino acids P≈G>F>Y≈W>T = N≈L are preferred in comparison to the cis conformation where C>>Q>P>S≈M>K≈G>V are favored in position i-2. For position i-1 the amino acids C>N>I = H>T = D≈L≈K≈Q≈R are predominant for the trans conformation while the order W>Y>>G>F>C>N>P≈Q≈A was found for the cis conformation. For the succeeding residue of Pro (residue i+1) the preferences E>Q = A≈D≈V = G≈R are observed for the trans conformation and the order Y≈ F>A>W = C>H>G≈S≈P = L≈Q resulted for the cis conformation. The most favorable amino acids are P≈W = G≈S≈M≈A≈F≈L for the trans conformation (although the highest ΔH value of P is only 1.09 indicating that this position has almost no preference for any amino acid) and P>C>>T>N>G≈R = K≈V for the cis conformation at position i+2.

By analyzing the individual positions, it appears that certain amino acids occur in the cis and in the trans sets with high propensity (in position i-2: G and P, in position i-1: C, N and Q, in position i+1: G, A and Q and in position i+2: P and G). Besides those amino acids a number of amino acids occur more exclusively at distinct positions for either the cis or the trans conformation. Interestingly, certain properties seem to dominate at particular positions. For cis, aromatic residues are very likely at position i-1 and i+1 whereas for trans aromatic residues occur with high propensity at position i-2.

In Table (5) the occurrences of the secondary structure types of proline in the cis and trans conformation are shown. For the five secondary structure elements (*bend*, *coil*, *helix*, *strand and turn*) we analyzed the surrounding of the fixed proline in the mid-position. Rows indicate the secondary structure type with fixed secondary structure of the proline. Columns show the relative occurrence of the secondary structure at the 4 positions around proline. Based on the relative occurrence of the secondary structure of the proline we can specify typical secondary structure pattern for cis and trans Xaa-Pro peptide bond conformation.

For example, if the cis proline is located in a bend or coil secondary structure type the amino acid at the position i-1 is never an element of a helix or turn. Furthermore, in the rare case where cis proline is part of a helix it is never found at the beginning or the end of the helix. In the case of the cis proline being present in a turn we never found a helix or bend structure element at the position i-1. However, residues in a turn occurred relatively often (378 times at the position i-1).

The 10 most frequently occurring secondary structure fragments with a cis proline at the mid position are presented in the second and third column of Table (6). For comparison, the occurrences of the corresponding fragments with trans proline are shown in column 4 and 5. In contrast, Table (7) shows the 10 most frequently occurring secondary structure fragments of trans proline and the corresponding occurrence of cis prolines. Here, the amount of helical regions in the environment of proline increases dramatically whereas in the cis case the helix structure occurs very rarely. Most of the trans prolines of the five-piece fragments in helical regions appear as the first helix element in contrast to the helical cis prolines which do never occur as the first element of a helix (Table (5)).

All previous results from the different approaches are compared with the content of the "Top 10" tables (Tables (6)(7)) in order to confirm the observed dependencies of sequence and secondary structure on the cis/trans peptide bond conformation of proline.

As a control parameter for the estimation of cis and trans conformations the sum of the Chou-Fasman parameter can be used (Table (1) and (2)). The sum of the  at the positions (i-1, i, i+1) leads to the same ranking in comparison to the "TOP 10" tables considering only 3 positions. For the pattern bbc in the cis case the sum is 7.746 and for trans conformation it is 2.541. In contrast the pattern ccc leads to 3.071 for trans and 2.275 for the cis conformation. On the basis of separated data sets for cis and trans proline conformation we derived the occurrence for the secondary structure elements and sequence pattern from so-called occurrence matrices (Table (3)(4) and (5)).

The output from the "TOP 10" tables can be verified in the occurrence matrices (Table (5)). The cbbcc pattern is most probable for the cis conformation and for trans conformation it is the ccccc pattern. They coincide as most probable secondary structure combination in Table (5) as highlighted by bold face. For example, for the secondary structure element bend in position i, ccbbc results as most probable for the trans proline and the cbbcc pattern for cis. For coil as secondary structure element in the position i ccccs results for the cis case whereas ccccc occurs for trans.

In order to evaluate the significance of Table (3) and (4), we analyzed the ccccc secondary structure pattern for the middle proline in trans peptide bond conformation in terms of the most probable amino acid in the 4 positions (H > 1). We found the following preferences: position i-2: P>M>Q>A>Y>T>R>S, position i-1: P>S>A>Q>R>K>M>C>L>T, position i+1: P>R>T>E>S>Q>A>V>K position i+2: P>S>K>R>Y>A>V>E. However, if those amino acids are compared with the most probable amino acids for trans or cis peptide bond conformation with proline in position i, no unambiguous sequence pattern remains.

We conclude that the influence of the secondary structure on a possible prediction of the Xaa-Pro peptide bond conformation is more significant than the sequence information of the residues surrounding proline.

### Analysis of the solvent accessible surface

The relationship between the relative solvent accessibility and the relative occurrence of prolines in our PDB data set was investigated. In the range from 0% to 20% of solvent accessibility 62.7% of the trans entries can be found compared to 56.1% cis entries whereas 43.9% cis instances occur in the range above the threshold of 20 % accessibility rate in contrast to 37.3% trans. These numbers suggest that proline in the cis conformation is slightly more frequently found in surface accessible areas compared to the trans proline.

The reason for the difference can be explained by the finding that cis prolines are more frequently found in solvent-exposed turn and bend structures, whereas trans prolines mostly occur in either helix or strand secondary structure elements. The relatively high frequency of exposed cis-prolines in conjunction with the relatively low energy barrier for the cis to trans conversion as compared to other amino acids mark proline as a preferred site for conformational switch mechanism in proteins. For example, PPIases catalyze the isomerization rate and thereby may regulate biological responses by alternative conformations of loop regions [14].

## Conclusion

In this work, we have analyzed more than 15000 proline residues within PDB-deposited protein structures in regard to their peptide bond conformation (cis or trans). We extracted fragments of 5 residues in length with proline in the mid-position. The PDB-derived secondary structure and the sequence information were used in a further statistical analysis of the 15778 fragments.

The calculation and interpretation of occurrence matrices reveal distinct preferences for the cis and for the trans conformation in dependence of secondary structure types. By the use of the modified Chou-Fasman parameter at the positions (i-1), (i) and (i+1) (equations 1–3) propensities for the proline peptide bond conformation can be derived from the secondary structure pattern. It is conceivable that an implementation of the modified Chou-Fasman parameters can be used for the prediction of proline peptide bond conformation in fragments with known secondary structure. An application of the new Chou-Fasman parameters for other naturally occurring amino acids is possible and leads to statistically relevant propensities for the prediction of the peptide conformation of any of the 20 amino acids in proteins. A prediction algorithm is presented on our website .

Finally the relationship between the relative solvent accessibility of proline and its peptide bond conformation shows that cis prolines occur more frequently in surface accessible areas compared to the prolines in trans conformation.

## Methods

### Materials

For data acquisition the PDB [15] was used by iterating over those protein entries who's PDB IDs are in a set of 3722 nonredundant proteins. All these proteins fulfill the following conditions: they share a maximum sequence identity of 25%, they have been solved to a resolution of 4.0 Å or less, they display a maximum R-value of 1.0 and a maximal chain length of 10000 amino acids (given by the PISCES [16] protein sequence culling service at .

From these proteins the coordinate section was extracted to calculate the Ω dihedral angle between adjacent residues. A Perl script using the angle calculation algorithm of the PDB tool *dihedrl.for *was used for this calculation. A peptide bond was defined to be in cis conformation if the Ω angle was between -30° and +30° whereas angles outside of this range are assumed to be trans. The resulting file contains the PDB ID, the chain notation, the position of the cis residue in the sequence, its amino acid three letter code and the calculated Ω angle.

The resulting set of the calculation comprised 954 proteins containing at least one peptide backbone conformation of cis. The adjacent residues and the secondary structure were extracted from the locally installed PDB files *pdb_seqres.txt *and *ss.txt*. In this way fragments of five amino acids were created with two residues flanking the proline at the mid-position on each side. The secondary structure of these five-residue segments were derived from the ss.txt of the respective PDB file and was calculated on the basis of the hydrogen bonding pattern by DSSP [17] denoting H as helix, B as beta bridge, E as strand, G as 3.1 helix, I as pi-helix, T as turn, S as bend and a blank space as coil structure. We grouped the DSSP derived structural information into the following five types: {*b*(end) = S, *c*(oil) = {B, }, *h*(elix) = {H, G, I}, *s*(trand) = E, *t*(urn) = T}. The resulting data set contained 15778 entries including 1390 fragments containing cis prolines. Each entry of the data set comprised the amino acid types at each position, the secondary structure information and the classification (cis/trans) (for example "R, S, P, F, T, c, b, b, c, t, cis").

### Chou-Fasman parameter

The correlation between secondary structure type and the conformation of proline can be calculated from the Chou Fasman parameters. We have applied the Chou-Fasman algorithm to elucidate this correlation by using the following formulas:







where *f*_*s *_denotes the occurrence of a certain secondary structure type with proline whether in the cis or trans conformation relative to the total occurrence of the same structure type. *f*_*class *_is the relation of the number of prolines in a specific conformation and the total number of prolines in the data set.  then describes the altered Chou-Fasman parameter for the probability of the cis or trans conformation of proline to be present in the individual secondary structure types.

### Solvent accessible area

The solvent accessible area was calculated for the whole protein with DSSP [17]. The DSSP algorithm provides information of the secondary structure based on the hydrogen bond pattern and of the solvent accessible area [18] of the different amino acids in the sequences. For the normalization of the accessible surface area of the prolines, the values obtained by DSSP were divided by the maximum solvent accessible area of an isolated proline residue (269 A^2^). The calculated relative accessibilities were in the range from 0% to 78%.

## Authors' contributions

DP, DLe, DLa, CF are responsible for data mining, statistical analysis and the manuscript preparation. DP & DLa programmed the scripts. All authors read and approved the final manuscript.

## Figures and Tables

**Table 1:** Chou-Fasman parameters (i). Chou-Fasman parameters for the cis/trans classes at position i regarding the secondary structure of proline.

Structure	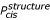	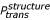
Bend	4.249	0.683
Coil	0.389	1.060
Helix	0.036	1.094
Strand	0.051	1.093
Turn	1.452	0.956

**Table 2:** Chou-Fasman parameters (i-1) (i+1). Chou-Fasman parameters for the cis/trans classes regarding the secondary structure of proline's predecessors and successors.

structure	*i-1*		*i+1*	
	
	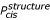	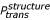	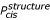	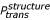
bend	2.054	0.897	1.263	0.974
coil	0.462	1.052	1.437	0.957
helix	0.086	1.089	0.321	1.066
strand	0.794	1.020	1.207	0.980
turn	2.261	0.877	0.786	1.021

**Table 3:** Occurrence matrix. Occurrence matrix for the amino acid combinations of trans and cis proline in the fixed position (i). The total number *N*_*total *_is the occurrence of an amino acid in all investigated sequence fragments. The number of the proline at the position (i) in the trans conformation is 14388 and in the cis conformation it is 1390. The absolute and relative occurrence of one amino acid is shown at all positions (rel and abs). The relative occurrence is the relation of the number of the amino acid at the position to the total number of all amino acid at this position.

			*trans Pro*								*cis Pro*							
			
			*Position*								*position*							
			
AA	Total		*i-2*		*i-1*		*i+1*		*i+2*		*i-2*		*i-1*		*i+1*		*i+2*	
	abs	rel	*abs*	*rel*	*abs*	*rel*	*abs*	*rel*	*abs*	*rel*	*abs*	*rel*	*abs*	*rel*	*abs*	*rel*	*abs*	*rel*

A:	4343	0.075	1024	0.071	1030	0.072	1169	0.081	1120	0.078	90	0.065	111	0.080	134	0.096	79	0.057
C:	810	0.014	204	0.014	256	0.018	166	0.012	184	0.013	38	0.027	25	0.018	24	0.017	33	0.024
D:	3565	0.062	818	0.057	972	0.068	956	0.066	819	0.057	57	0.041	59	0.042	52	0.037	83	0.060
E:	3604	0.063	799	0.056	685	0.048	1208	0.084	912	0.063	70	0.050	84	0.060	47	0.034	61	0.044
F:	2426	0.042	656	0.046	562	0.039	596	0.041	612	0.043	59	0.042	82	0.059	96	0.069	58	0.042
G:	4275	0.074	1202	0.084	824	0.057	1113	0.077	1136	0.079	112	0.081	154	0.111	112	0.081	106	0.076
H:	1418	0.025	323	0.022	418	0.029	313	0.022	364	0.025	32	0.023	29	0.021	40	0.029	32	0.023
I:	3178	0.055	780	0.054	915	0.064	711	0.049	772	0.054	68	0.049	38	0.027	54	0.039	62	0.045
K:	2804	0.049	654	0.045	745	0.052	703	0.049	702	0.049	76	0.055	66	0.047	57	0.041	70	0.050
L:	5279	0.092	1336	0.093	1423	0.099	1179	0.082	1341	0.093	96	0.069	90	0.065	134	0.096	96	0.069
M:	1131	0.020	292	0.020	272	0.019	263	0.018	304	0.021	35	0.025	21	0.015	22	0.016	28	0.020
N:	2683	0.047	684	0.048	816	0.057	608	0.042	575	0.040	65	0.047	79	0.057	56	0.040	70	0.050
P:	3121	0.054	896	0.062	687	0.048	691	0.048	847	0.059	98	0.071	82	0.059	78	0.056	149	0.107
Q:	2105	0.037	480	0.033	556	0.039	574	0.040	495	0.034	72	0.052	56	0.040	53	0.038	45	0.032
R:	2591	0.045	644	0.045	675	0.047	655	0.046	617	0.043	61	0.044	49	0.035	54	0.039	64	0.046
S:	3596	0.062	886	0.062	887	0.062	877	0.061	946	0.066	108	0.078	84	0.060	91	0.065	79	0.057
T:	3479	0.060	876	0.061	948	0.066	818	0.057	837	0.058	84	0.060	61	0.044	77	0.055	102	0.073
V:	4165	0.072	1021	0.071	1036	0.072	1073	0.075	1035	0.072	101	0.073	65	0.047	97	0.070	101	0.073
W:	815	0.014	221	0.015	169	0.012	206	0.014	219	0.015	18	0.013	44	0.032	23	0.017	19	0.014
Y:	2164	0.038	592	0.041	512	0.036	509	0.035	551	0.038	50	0.036	111	0.080	89	0.064	53	0.038

**Table 4:** Normalized relative occurrences. Normalized relative occurrences of all amino acids at the different positions. For the normalization we used the coefficient of each observed occurrence and the natural occurrence for each amino acid at each position. Highlighted in bold are the most probable amino acid (H > 1) occurring at the four positions.

AA	*H*_*trans *_*i-2*	*H*_*cis *_*i-2*	*H*_*trans *_*i-1*	*H*_*cis *_*i-1*	*H*_*trans *_*i+1*	*H*_*cis *_*i+1*	*H*_*trans *_*i+2*	*H*_*cis *_*i+2*
A:	0.95	0.87	0.96	**1.07**	**1.08**	**1.28**	**1.04**	0.76
C:	1.00	**1.93**	**1.29**	**1.29**	0.86	**1.21**	0.93	**1.71**
D:	0.92	0.66	**1.10**	0.68	**1.06**	0.60	0.92	0.97
E:	0.89	0.79	0.76	0.95	**1.33**	0.54	1.00	0.70
F:	**1.10**	1.00	0.93	**1.40**	0.98	**1.64**	**1.02**	1.00
G:	**1.14**	**1.09**	0.77	**1.50**	**1.04**	**1.09**	**1.07**	**1.03**
H:	0.88	0.92	**1.16**	0.84	0.88	**1.16**	1.00	0.92
I:	0.98	0.89	**1.16**	0.49	0.89	0.71	0.98	0.82
K:	0.92	**1.12**	**1.06**	0.96	1.00	0.84	1.00	**1.02**
L:	**1.01**	0.75	**1.08**	0.71	0.89	**1.04**	**1.01**	0.75
M:	1.00	**1.25**	0.95	0.75	0.90	0.80	**1.05**	1.00
N:	**1.02**	1.00	**1.21**	**1.21**	0.89	0.85	0.85	**1.06**
P:	**1.15**	**1.31**	0.89	**1.09**	0.89	**1.04**	**1.09**	**1.98**
Q:	0.89	**1.41**	**1.05**	**1.08**	**1.08**	**1.03**	0.92	0.86
R:	1.00	0.98	**1.04**	0.78	**1.02**	0.87	0.96	**1.02**
S:	1.00	**1.26**	1.00	0.97	0.98	**1.05**	**1.06**	0.92
T:	**1.02**	1.00	**1.10**	0.73	0.95	0.92	0.97	**1.22**
V:	0.99	**1.01**	1.00	0.65	**1.04**	0.97	1.00	**1.01**
W:	**1.07**	0.93	0.86	**2.29**	1.00	**1.21**	**1.07**	1.00
Y:	**1.08**	0.95	0.95	**2.11**	0.92	**1.68**	1.00	1.00

**Table 5:** Secondary structure matrix. The occurrence of the secondary structure types of proline in the cis and trans conformation is shown. For the five defined secondary structure elements (*bend*, *coil*, *helix*, *strand and turn*) we analyzed the surrounding of the fixed proline in the mid-position. Rows indicate the secondary structure type with fixed secondary structure of the proline. Columns show the relative occurrences of the secondary structure at the 4 positions around proline. Bold face highlights the most probable secondary structure. For the cis peptide bond conformation of the central proline in helix and strand not enough entries could be collected from the PDB to reach significance.

secondary *structure*	structure of trans Pro									structure of cis Pro								
	
	position									position								
	
	*i-2*		*i-1*		*bend*	*i+1*		*i+2*		*i-2*		*i-1*		*bend*	*i+1*		*i+2*	
bend:	257	0.21	317	0.26	1211	684	**0.56**	229	0.19	176	0.24	467	**0.63**	742	143	0.19	105	0.14
coil:	462	**0.38**	813	**0.67**	1211	334	0.28	545	**0.45**	329	**0.44**	180	0.24	742	433	**0.58**	321	**0.43**
helix:	77	0.06	2	0.00	1211	22	0.02	107	0.09	31	0.04	0	0.00	742	28	0.04	70	0.09
strand:	254	0.21	74	0.06	1211	135	0.11	228	0.19	164	0.22	95	0.13	742	104	0.14	174	0.23
turn:	161	0.13	5	0.00	1211	36	0.03	102	0.08	42	0.06	0	0.00	742	34	0.05	72	0.10
	*i-2*		*i-1*		*coil*	*i+1*		*i+2*		*i-2*		*i-1*		*coil*	*i+1*		*i+2*	
bend:	1401	0.22	1614	0.25	6505	952	0.15	901	0.14	41	0.18	80	0.35	231	29	0.13	37	0.16
coil:	2966	**0.46**	4430	**0.68**	6505	3749	**0.58**	2656	**0.41**	112	**0.48**	137	**0.59**	231	127	**0.55**	81	0.35
helix:	377	0.06	8	0.00	6505	532	0.08	862	0.13	9	0.04	0	0.00	231	9	0.04	14	0.06
strand:	934	0.14	360	0.06	6505	788	0.12	1312	0.20	41	0.18	13	0.06	231	62	0.27	91	**0.39**
turn:	827	0.13	93	0.01	6505	484	0.07	774	0.12	28	0.12	1	0.00	231	4	0.02	8	0.03
	*i-2*		*i-1*		*helix*	*i+1*		*i+2*		*i-2*		*i-1*		*helix*	*i+1*		*i+2*	
bend:	361	0.14	193	0.07	2603	1	0.00	12	0.00	0	0.00	0	0.00	8	0	0.00	1	0.13
coil:	752	**0.29**	913	**0.35**	2603	1	0.00	38	0.01	0	0.00	0	0.00	8	0	0.00	2	0.25
helix:	722	**0.28**	935	**0.36**	2603	2597	**1.00**	2489	**0.96**	8	1.00	8	1.00	8	8	1.00	0	0.00
strand:	165	0.06	43	0.02	2603	0	0.00	6	0.00	0	0.00	0	0.00	8	0	0.00	0	0.00
turn:	603	0.23	519	0.20	2603	4	0.00	58	0.02	0	0.00	0	0.00	8	0	0.00	5	0.63
	*i-2*		*i-1*		*strand*	*i+1*		*i+2*		*i-2*		*i-1*		*strand*	*i+1*		*i+2*	
bend:	124	0.09	98	0.07	1352	123	0.09	202	0.15	0	0.00	2	0.22	9	1	0.11	1	0.11
coil:	268	0.20	157	0.12	1352	391	0.29	322	0.24	4	0.44	2	0.22	9	2	0.22	2	0.22
helix:	10	0.01	0	0.00	1352	80	0.06	115	0.09	0	0.00	0	0.00	9	0	0.00	0	0.00
strand:	788	**0.58**	1096	**0.81**	1352	703	**0.52**	603	**0.45**	3	0.33	4	0.44	9	6	0.67	4	0.44
turn:	162	0.12	1	0.00	1352	55	0.04	110	0.08	2	0.22	1	0.11	9	0	0.00	2	0.22
	*i-2*		*i-1*		*turn*	*i+1*		*i+2*		*i-2*		*i-1*		*turn*	*i+1*		*i+2*	
bend:	334	0.12	247	0.09	2717	12	0.00	450	0.17	57	0.14	0	0.00	400	54	0.14	39	0.10
coil:	777	**0.29**	1266	**0.47**	2717	26	0.01	835	**0.31**	142	**0.36**	9	0.02	400	88	0.22	138	**0.35**
helix:	534	0.20	127	0.05	2717	239	0.09	414	0.15	34	0.09	0	0.00	400	57	0.14	74	0.19
strand:	447	0.16	155	0.06	2717	20	0.01	192	0.07	38	0.10	13	0.03	400	20	0.05	69	0.17
turn:	625	0.23	922	0.34	2717	2420	**0.89**	826	**0.30**	129	0.32	378	**0.95**	400	181	**0.45**	80	0.20

**Table 6:** 10 most frequent cis secondary structure combinations. Comparison of the 10 most frequent cis secondary structure combinations versus the corresponding trans secondary structure combinations.

Structure	cis Pro		Trans Pro	
	
	absolute	relative(%)	absolute	relative(%)
cbbcc	93	6.6906	24	0.1668
bbbcc	58	4.1727	28	0.1946
cbbss	45	3.2374	7	0.0487
cttcc	31	2.2302	3	0.0209
ssbcc	30	2.1583	12	0.0834
ttttt	28	2.0144	166	1.1537
ccbcc	25	1.7986	48	0.3336
cbbbc	25	1.7986	22	0.1529
bbbss	25	1.7986	21	0.146
sbbcc	24	1.7266	5	0.0348

**Table 7:** 10 most frequent trans secondary structure. Comparison of the 10 most frequent trans secondary structure combinations versus the corresponding cis secondary structure combinations.

structure	trans Pro		Cis Pro	
	
	absolute	relative(%)	absolute	relative(%)
ccccc	1311	9.1118	23	1.6547
cchhh	524	3.6419	0	0
hhhhh	398	2.7662	0	0
tthhh	291	2.0225	0	0
ccttc	251	1.7445	1	0.0719
cccss	241	1.675	9	0.6475
bbccc	221	1.536	5	0.3597
hthhh	218	1.5152	0	0
ccchh	213	1.4804	3	0.2158
ccttt	210	1.4595	2	0.1439
